# Assembly of the *Candida albicans *genome into sixteen supercontigs aligned on the eight chromosomes

**DOI:** 10.1186/gb-2007-8-4-r52

**Published:** 2007-04-09

**Authors:** Marco van het Hoog, Timothy J Rast, Mikhail Martchenko, Suzanne Grindle, Daniel Dignard, Hervé Hogues, Christine Cuomo, Matthew Berriman, Stewart Scherer, BB Magee, Malcolm Whiteway, Hiroji Chibana, André Nantel, PT Magee

**Affiliations:** 1 Biotechnology Research Institute, National Research Council of Canada, Montreal, Quebec, H4P 2R2, Canada; 2 University of Minnesota, Minneapolis, MN, 55455, USA; 3 Broad Institute of MIT and Harvard, Cambridge, MA, USA; 4 Wellcome Trust Sanger Institute, Hinxton, CB10 1SA, UK; 5 Paseo Grande, Moraga, CA 94556, USA; 6 Research Center for Pathogenic Fungi and Microbial Toxicoses, Chiba University, Chiba, 260-8673, Japan

## Abstract

**Background:**

The 10.9× genomic sequence of *Candida albicans*, the most important human fungal pathogen, was published in 2004. Assembly 19 consisted of 412 supercontigs, of which 266 were a haploid set, since this fungus is diploid and contains an extensive degree of heterozygosity but lacks a complete sexual cycle. However, sequences of specific chromosomes were not determined.

**Results:**

Supercontigs from Assembly 19 (183, representing 98.4% of the sequence) were assigned to individual chromosomes purified by pulse-field gel electrophoresis and hybridized to DNA microarrays. Nine Assembly 19 supercontigs were found to contain markers from two different chromosomes. Assembly 21 contains the sequence of each of the eight chromosomes and was determined using a synteny analysis with preliminary versions of the *Candida dubliniensis *genome assembly, bioinformatics, a sequence tagged site (STS) map of overlapping fosmid clones, and an optical map. The orientation and order of the contigs on each chromosome, repeat regions too large to be covered by a sequence run, such as the ribosomal DNA cluster and the major repeat sequence, and telomere placement were determined using the STS map. Sequence gaps were closed by PCR and sequencing of the products. The overall assembly was compared to an optical map; this identified some misassembled contigs and gave a size estimate for each chromosome.

**Conclusion:**

Assembly 21 reveals an ancient chromosome fusion, a number of small internal duplications followed by inversions, and a subtelomeric arrangement, including a new gene family, the *TLO *genes. Correlations of position with relatedness of gene families imply a novel method of dispersion. The sequence of the individual chromosomes of *C. albicans *raises interesting biological questions about gene family creation and dispersion, subtelomere organization, and chromosome evolution.

## Abstract 2

For Assembly 20 of the *Candida albicans *genome, the sequence of each of the eight chromosomes was determined, revealing new insights into gene family creation and dispersion, subtelomere organization, and chromosome evolution.

## Background

In the past 25 years, the opportunistic human pathogen *Candida albicans *has become a serious medical problem. This fungus is now fourth on the list of hospital-acquired infections, ahead of Gram-negative bacteria, and despite the recent introduction of a new class of antifungals, drug resistance continues to be a problem [1]. In the past 15 years, molecular techniques have been applied to understand the pathogenesis of this organism as well as to search for novel drug targets. However, *C. albicans *presents several difficulties for molecular biologists: it is diploid; only a part of a sexual cycle has been demonstrated; it has a very plastic genome; and it is highly heterozygous. Each of these properties is best investigated through a genomic approach. Hence, knowledge of the genome sequence has been an important goal for the past 10 years. More recently, genome structure and dynamics have become increasingly important in this organism as widespread aneuploidy [2,3], the role of repeated DNA in chromosome loss [4], and chromosome rearrangement leading to drug resistance [5] have been reported.

The Candida Genome Sequencing Project started in 1996 and, in 2004, it produced a diploid assembly constructed from 10.9× coverage (Assembly 19), which provided single contigs where heterozygosity was not obvious and allelic contigs where there was significant heterozygosity [6]. There were several important steps along the way to this release; these are detailed in a review by Nantel [7]. The first was the construction of a physical map of one chromosome, chromosome 7 [8]. Next were the two early releases of the emerging sequence data, called Assembly 4 and Assembly 6. These lower density assemblies facilitated a great deal of gene analysis, including the construction of several microarrays [9,10], an analysis of haploinsufficient genes for filamentation [11], and the elucidation of several gene families, including a number important in pathogenesis. Examples include the secreted aspartyl proteinases (*SAP*s) [12], the agglutinin-like substances (*ALS*s) [13], and the phospholipases (*PLB *and *PLC*) [14,15]. Two quite comprehensive disruption libraries are currently available. One library was constructed systematically by targeted disruption of one allele followed by insertion of a regulated promoter at the other allele [16]. The other disruption library was constructed randomly by transposon mutagenesis, using as an insert into one allele the UAU cassette, which facilitates disruption of the second allele via two spontaneously occurring steps of mitotic recombination [17].

These tools have greatly advanced the pace of molecular analysis of the pathogenesis and life style of *C. albicans*, but Assembly 19 was not a finished sequence, since it contained a total of 412 contigs, of which 266 were the haploid set. In order to provide a finished sequence, we used hybridization of chromosomes partially purified by pulse-field elecrophoresis as well as a sequence tagged site (STS) map based on a fosmid library to identify the chromosomal location of various contigs. We then utilized bioinformatics to analyze both the emerging sequence of *C. albicans *strain WO-1, the sister species *Candida dubliniensis *and the primary traces used to generate Assembly 4, and coupled this with the STS map and a whole-chromosome optical map to construct Assembly 21. This assembly has eight linear DNA sequences including nine copies of the intermediate repeat called the major repeat sequence (MRS), of which three have been completely sequenced. The MRS is made up of three subrepeats, called RB2, RPS, and HOK [18]. In addition to the intact MRS sequences, there are 14 RB2 sequences and 2 HOK sequences. The ribosomal DNA constitutes another repeat, which is not included in the assembly. In addition to its usefulness for gene mapping, Assembly 21 reveals some interesting biological features, including a putative transcription factor gene family with members proximal to 14 of the 16 telomeres, a telomere-like sequence in the middle of chromosome 1, information on the relationships of chromosome location to similarity of gene families, and a revised open reading frame (ORF) list.

## Results

### Assembly 21

The completed Assembly 21 contains 15.845 Mb of DNA, organized into the 8 *C. albicans *chromosomes. The assembly does not include the complete telomeric sequences for every chromosome, and includes only one copy of the normally repeated rDNA on chromosome R. All but two chromosomes end with the subtelomeric repeat CARE-2 (Rel-2) [19,20] at each telomere. This repeat was originally shown to be telomere-associated on chromosome 7 [8]. Macro-restriction maps locate MRSs on all chromosomes but chromosome 3, but since the MRSs are highly repeated and more than 16 kb in size, they are represented but not included in the assembly. On chromosomes 7 and 6, where the MRSs have been sequenced, they are inserted into the sequence. The finished sequence of chromosome 7 has been published elsewhere [21]; this assembly includes a slightly revised version. In addition to the missing MRS regions there is one gap, on chromosome 3. Assembly 21 is a haploid assembly; in cases where Assembly 19 detected heterozygosity, allelic contigs 19-1XXXX and 19.2XXXX were assembled. In regions where there were allelic contigs in Assembly 19, only the 19-1XXXX contigs were used to construct Assembly 21, so it provides no information about heterozygosity.

### Chromosome size and structure

Table 1 shows the sizes of the individual chromosomes in Assembly 21 and compares the size of the sequence with the size of each chromosome as determined by the optical map. The Assembly 21 size in Table 1 does not include the MRS if one or more are present, and for chromosome R, only one copy of the rDNA repeat is included. The chromosomes range in size from 3,190,598 bases for chromosome 1 to 943,480 bases for chromosome 7. For chromosome R, the actual number of rDNA repeat adds 350 kb to one homologue and 800 kb to the other. Where chromosome homologues are of different sizes, as with chromosome R, the optical map software will choose one homologue. The optical map thus gives the size, including the rDNA, of the smaller homologue of chromosome R. The larger homologue is very close to chromosome 1 in size, about 3.1 Mb. The fact that the subtelomeric repeat CARE-2 is missing from one telomere may indicate that the sequence does not extend to the end of the chromosome. On chromosomes 2 and 7 both the *TLO *gene and the CARE-2 sequences are missing. We were able to map 233,091 out of 250,884 (93%) of the original sequence traces on Assembly 21. The remaining 17,793 sequences probably represent some of these missing sequences as well as allelic variants but it was impossible to map them to the current assembly.

Assembly 21 demonstrates that the sub-telomeric repeats found on chromosome 7 [21] are characteristic of all the chromosomes. The common factor is all or part of the repeat CARE-2 [19], which includes the long terminal repeat (LTR) kappa (AF041469) and shares some sequence with Rel-2 [20]. There are several other LTRs at the telomeres, and on chromosome 1R there is an intact transposon pCa1 (AF007776). Although one telomere on each of chromosomes 2 and 7 in Assembly 21 is missing CARE-2 (and the *TLO *gene (see below)), the fosmid map shows these repeats on every telomere. Sequence near the telomere is hard to clone, and the most likely interpretation of this discrepancy is that the appropriate clones for these regions were underrepresented in the Stanford library. Since the repeats tend to be telomere-proximal to the *TLO *gene, the fact that *TLO *is missing on chromosomes 2 and 7 also suggests that some sequence is missing. The detailed organization of the sub-telomeric repeats is complex and differs at each telomere.

### Centromeres

Sequences that bind the Cse4p protein (the CENP-A orthologue) from *C. albicans *have been identified on each chromosome by Sanyal *et al*. [22], who suggested that they are at least part of the centromere. This interpretation is supported by the observation that the sequence identified on chromosome 7 contributes to its mitotic stability. Table 1 gives the address (distance in bases from the left-hand end of the chromosome) of each centromere and shows that the chromosomes are generally metacentric, with the centromere sequences located near the center of the chromosomes. However, chromosomes 2 and 6 are acrocentric. On chromosome 2 the centromere is about 85% of the way toward the right telomere, and on 6 it is more than 95%. In contrast to *Saccharomyces cerevisiae *and *Candida glabrata*, there are no sub-telomeric gene families other then the *TLO *genes in *C. albicans*.

### ORF analysis

The ORF analysis of Assembly 21 is being carried out at the Candida Genome Database (47). The present analysis, based on the previous assembly, 20, differs slightly from that of Assembly 19. The human-curated annotation of Assembly 19 identified 6,354 genes. The Candida Genome Database contains 12,015 genes, including allelic variants. Assembly 20 contains 6,090 genes. Of these, the identity and sequence of 6,065 have not changed significantly from Assembly 19 (>98% sequence identity). Fourteen new ORFs have been added to Assembly 20. Thus, 290 genes from the annotation of Assembly 19 are not in Assembly 20. Of these, 192 have been found to be identical to other Assembly 20 genes and have >90% of their sequence incorporated in these genes, while an additional 19 have >50% of their sequence incorporated into another ORF. The 79 remaining Assembly 19 ORFs have no strong Blast hits and include 55 hypotheticals, 15 gene family members, 7 that were truncated, 1 that was overlapping, and 2 putative sequencing errors. The Candida Genome Database provides an extensive analysis of the changes in ORF classifications between the two assemblies. Some discrepancies between their numbers and ours arise from the fact that they started from a greater number of orf19 genes.

### Repeated DNA

*C. albicans *was shown to have eight chromosomes by pulse-field electrophoresis. Early studies demonstrated not only that the organism was diploid but that it contained several large blocks of repeated DNA, the MRS, with a complete or partial copy on each of the eight chromosomes [18,23-25].

Analysis of the emerging sequence demonstrated the existence of a large number of LTRs and other repeated sequences related to transposons [26]. Gene families also constitute a significant source of repeated DNA. Some of these repeated sequences, especially the MRSs, are too large to be crossed in a single sequencing run, so that assembly using bioinformatics is blocked. The sequences of the MRSs on chromosomes should be considered unreliable due to the repeated nature of MRSs, which attract traces from many different other MRSs in the genome. The smaller repeated DNA regions are subject to the same potential problem and led to erroneous assembly in some of the Assembly 19 contigs. The fosmid map and the optical map were very helpful in identifying these errors and correcting them.

### The *TLO *gene family

**Figure 1:**
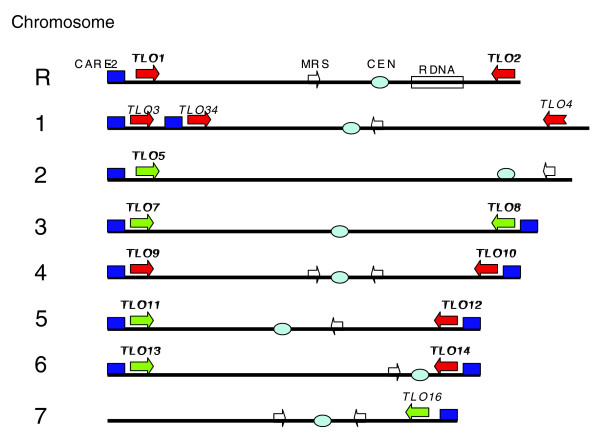
Schematic representation of the chromosomes in Assembly 21. The major structural features, including the centromeres (CEN), the MRS sequences, and the CARE-2 sequences are represented. The *TLO *genes without introns are shown in red; those with introns are in green. The positions are approximately to scale. The smaller homologue of chromosome R (containing a 350 kb rDNA repeat) is shown.

**Figure 2:**
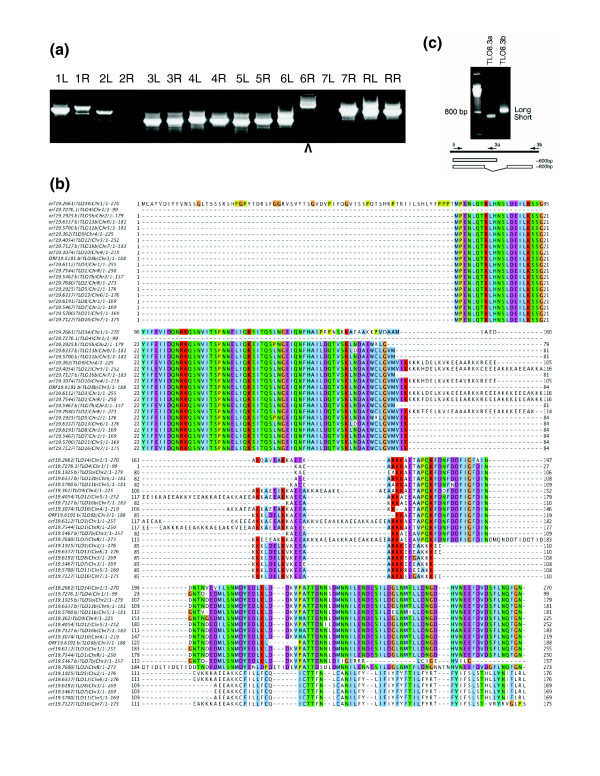
Analysis of the *TLO *gene family. **(a) **PCR validation of the telomeric *TLO *genes using a universal *TLO *primer coupled with a second primer that is specific for each chromosome arm. A putative *TLO14 *gene was identified on the 6R arm (see arrowhead). **(b) **Identification, by rtPCR, of two putative splicing variants using a 5' oligo that will recognize all *TLO *genes and two 3' primers with partial specificity for the two possible versions of the *TLO8 *transcripts. **(c) **Alignment of the amino acid sequences of the 14 *TLO *genes that are present in the latest genome assembly.

The putative transcription factor gene *CTA2 *was identified by Kaiser *et al*. [27] in a one-hybrid screen in *S. cerevisiae*. Goodwin and Poulter [26] first noticed that this or a related sequence was often telomere-associated. The *CTA2 *sequence has homology to members of a gene family found at 14 of the 16 telomeres. Since *CTA2 *is a gene name, we renamed the family *TLO *for TeLOmere-associated genes. On this assembly, they are numbered so that the left arm of the chromosome has an odd-numbered *TLO *gene and the right *TLO *gene has an even number, ascending from chromosome R through chromosome 7. Thus, chromosome R has *TLO1 *on the left and *TLO2 *on the right. Although the original member of this gene family was isolated because it has transactivating activity in *S. cerevisiae*, the function of these genes in *C. albicans *has not been determined. There are 15 members of this gene family in Assembly 21; 14 are located within 14 kb of an end of a chromosome contig, and all are oriented in a 5'-3' direction toward the centromere and away from the telomere (Figure 1). In addition, all but one (chromosome 1R) have the LTR kappa 5' to the ORF, usually immediately adjacent but sometimes a few kb away. One member of the *TLO *gene family (ORF19.2661) is found in the interior of chromosome 1, 1.29 Mb from the left end of this 3.19 Mb chromosome. Like the other family members, this ORF points toward the centromere and away from the telomere and has a kappa sequence from the subtelomeric repeat CARE-2 in its 5' region. In this case, the kappa sequence is the only part of the CARE-2 sequence present. Thus, this particular copy resembles all the telomere-associated family members but is located in the middle of chromosome 1, and we have numbered it *TLO34*, since it is located between *TLO3 *and *TLO4*. In order to keep the numbering system consistent, we have saved the names *TLO6 *and *TLO15 *for the genes on 2R and 7L that we expect will eventually be identified. In addition, Figure 2a demonstrates that *TLO14*, on the right arm of chromosome 6, has been confirmed by PCR but this gene has yet to be sequenced.

An alignment of the amino acid sequences encoded by 13 *TLO *genes is shown in Figure 2c. While we initially noticed that six of them (*TLO5*, *7*, *8*, *11*, *13*, and *16*) had a different carboxy-terminal domain, an astute reviewer remarked that adding an intron to the annotations of these genes would allow them to code for a protein with a carboxy-terminal end that is similar to the other *TLO *genes. We used rtPCR with a universal 5' primer and gene-specific 3' primers to show that both types of transcripts are expressed in *Candida *cells. Although the amplicons nevertheless contained a mixture of two or more alleles or family members, end sequencing clearly showed that the long form contained the common carboxy-terminal sequences while the short form encoded the unique sequences first noticed in *TLO5*. Although the annotation assumes that all six members of this TLO family sub-group yield transcripts in both spliced and unspliced forms, confirmation would require screening of an expressed sequence tag library. Finally, *TLO4 *(orf19.7276.1) lies almost at the edge of a sequence contig and is obviously missing an upstream exon while the additional amino-terminal domain encoded by *TLO34 *may or may not be part of the actual transcript.

### Other gene families

As noted by Braun and coworkers [28], *C. albicans *has a number of gene families. These range in size from 2 members to as many as 26 (the ABC transporter superfamily) [29]. Several are clustered on one or two chromosomes. For example, chromosome 6 contains members of six families, with five represented by more than one member, and one, *ALS*, with five members. Most of the families with more than two members have representatives on more than one chromosome. In addition, some two-member families are on different chromosomes. Examination of the genes surrounding family members on different chromosomes for the most part gives no hint as to the mechanism by which homologous sequences arrived at different locations.

**Figure 3:**
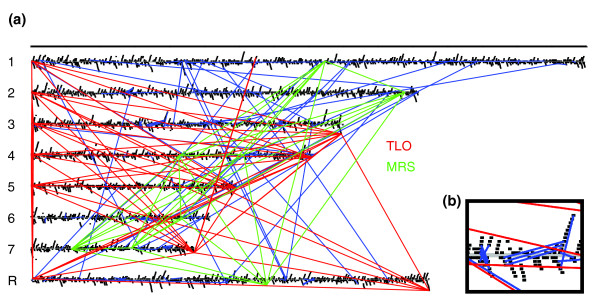
Highly related ORFs in the *C. albicans *genome. **(a) **The eight chromosomes are shown with the ORFs indicated by the small black lines. The red lines connect the *TLO *genes, while the green lines connect the ORFs in the MRS sequence. The blue lines connect ORFs with a relatedness greater than 80%. **(b) **Close-up of chromosome 3 from base 622,329 to base 785,321 showing two areas that appear to have undergone duplication followed by inversion.

The arrangement of these families on the chromosomes is not random. Figure 3a shows the ORFs from Assembly 21 aligned along the eight chromosomes. ORFs with 80% or more similarity are connected by lines. There are some areas that appear to be tandem duplications. An example from chromosome 3 is shown in Figure 3b. The sequence containing the gene *DTD2 *and those for two hypothetical proteins seem to have undergone a duplication accompanied by an inversion. A similar event seems to have occurred 40 kb away involving the genes *DAL5*, *ECM18*, and *FUR4*. There are no sequences such as LTRs near these duplications. Although the most plausible mechanism for the generation of gene families whose members are close together on one chromosome is tandem duplication, in many cases of gene families that contain tandemly repeated genes, the most closely related members are dispersed. Table 2 shows this for the *SAP *(Secreted Aspartyl Proteinase) and *LIP *(LIPase) gene families. For the *SAP *gene family, only *SAP5 *and *SAP6*, 84 kb apart on chromosome 6, are both nearest neighbors and most closely related in sequence. *SAP1 *and *SAP4 *are adjacent and each is most closely related to another member. For the *LIP *gene family, *LIP5 *is most similar to *LIP9*, and they are 12 kb apart on chromosome 7. Family members *LIP1*, *6*, and *10 *are adjacent on chromosome 1 and each is most homologous to a distal gene (*LIP3 *for *LIP1 *and *LIP2 *for *LIP6 *and *LIP 10*). It is interesting to note from Figure 3a that the number of highly similar ORFs outside the *TLO *and MRS sequences (represented by the blue connecting lines) is relatively small compared to the number of gene families.

## Discussion

Assembly 19, the diploid assembly of the genome of *C. albicans *strain SC5314 was a very important achievement. It provided a great deal of insight into many aspects of genomic organization, especially the large amount of heterozygosity [6]. The subsequent annotation of the assembly by the community demonstrated a number of important properties of the genome, including the number of genes (6,354), the number with introns (224), the frequency and characteristics of short tandem repeats, and the characteristics of several multigene families. Braun *et al*. [28] also identified putative spurious genes and genes either on overlapping contigs or truncated by the end of contigs. However, they did not address chromosome location nor try to join the 266 haploid contigs of Assembly 19 into chromosome-sized assemblies. Thus, although these two projects brought the *C. albicans *genome to a very useful state, they still left it incomplete, lacking chromosome-size contigs and with some genes in an ambiguous state. Subsequently, Chibana *et al*. [21] completed the sequence of chromosome 7, identified 404 genes, and compared the synteny to the *S. cerevisiae *genome. They sequenced the MRSs and the gaps left in Assembly 19. They then aligned the sequence on the chromosome as determined by the physical map [8].

We undertook to complete the assembly (on a haploid basis) of all the chromosomes of *C. albicans*. We ordered and aligned the existing contigs along the chromosomes, filled in the gaps either by reexamining the traces at the Stanford Genome Technology Center, by gap sequencing or by using the emerging *C. albicans *WO-1 sequence to correct two regions of chromosomes 1 and 4. The assembly was also based on the STS fosmid map and on an optical map. As completed, the assembly consists of 16 supercontigs, interrupted on 5 chromosomes only by large blocks of repeated DNA. The contigs for chromosomes 6 and 7, for which the MRSs have been sequenced, have no gaps, while chromosome 3 has one gap, where adjacent contigs could not be joined. Chromosome 4 has two gaps and 5, 2, and 1 have one gap each, corresponding to the MRS. Chromosome R has two gaps, one for the MRS and one for the rDNA. Thus, there is only one gap in the unique sequence of the *C. albicans *genome that cannot be filled with sequence data from either SC5314 or WO-1. We identified 85 junctions that could not be filled with the original SC5314 sequence traces. We successfully amplified 82 of these (see Additional data file 1) and work is continuing to amplify the last three junctions and to produce 'SC5314-pure' sequence. We have used this assembly to determine the size of the various chromosomes and to examine several unique aspects of the genome, including the subtelomeric regions, the gene families, and evidence for chromosome rearrangements. Our ultimate objectives are to identify aspects of the genome that affect virulence and to increase our understanding of the evolutionary mechanisms that affect the genome of this fungal pathogen.

There are chromosome size discrepancies between Assembly 21 and the optical map; these are attributable to several causes. Where the Assembly 21 size is smaller than the optical map size, the explanation may be the missing MRS, missing telomere-associated sequences, or size heterozygosity between the homologues. For example, we know that on chromosome 5 the MRS is 50 kb in size [4], very close to the difference between the two estimates. Where the size determined by the optical map is smaller (chromosomes 2, 3, and 4), the difference seems most likely to be heterozygosity for insertions of retrotransposon-related sequences. In these cases, the optical map of this chromosome is probably derived from the smaller homologue. For chromosome 2, the discrepancy is rather large, given that this chromosome in Assembly 21 lacks the MRS and probably some telomere sequences. Interestingly, the size estimates in Jones *et al*. [6] for the various chromosomes are remarkably close to the sizes determined by the optical map in Table 1.

One piece of information that comes out of our assembly is the similarity of the sequence of the *C. dubliniensis *genome to that of *C. albicans*. Although the karyotypes of these two organisms are quite divergent, the arrangement of genes within the chromosomes is similar enough to be of great assistance in mapping the contigs from Assembly 19. This bears out the evidence from the presence of MRS-like sequences and the ability to produce interspecies hybrids [30] that these two species are very closely related indeed. Other studies have shown that only about 4.4% (247) of *C. albicans *genes have less than 60% homology to *C. dubliniensis *[31]. Our results suggest that intergenic regions also show regions of significant sequence conservation.

The amount of repeated DNA in *C. albicans *is significant. The MRSs were a major problem and their placement on the chromosomes required the physical and optical maps. Chromosomes 4 and 7 each have two MRSs forming an inverted repeat, and in principle the internal DNA fragment could invert via mitotic recombination. In strain SC5314 and its derivative, CAI-4, this inversion seems to occur very rarely, at least in the laboratory. In spite of the fact that most of the known translocations in *C. albicans *occur at the MRS, suggesting that this is a hot spot for recombination, there is no evidence on either chromosome for a flip of the bracketed sequence.

The specific sequences of six of the nine MRSs are unavailable. This is only a problem if sequence variation in the MRS plays a biological role, and there is no evidence that it does. In addition to the MRSs and the subtelomeric repeats, there are more than 350 LTR sequences belonging to 34 different families scattered throughout the genome [26], and several of these are found clustered at telomeres. The subtelomeric repeat CARE-2 contains an LTR called kappa [32], which is found at the 5' end of each member of the *TLO *gene family. Whether this is related to the expansion of this family to the telomeres is not clear. The repeated DNA led to misassembly of some contigs in Assembly 19, including chimeras, artifactual duplications, and omitted sequence. The two physical maps and the *C. dubliniensis *sequence were essential in sorting out these artifacts.

The numerous gene families in *C. albicans *distinguish it from *S. cerevisiae*. A very common feature of these families is a clustering of members on a particular chromosome, which might reflect an ontology wherein a single copy undergoes tandem duplication and then sequences diverge as function diverges. There are several instances where similar but oppositely oriented gene clusters suggest that an inverted duplication of a region larger than a gene has occurred (Figure 3a).

The model for gene family ontology of duplication followed by dispersion would predict that, in general, similarity should be related to proximity. The arrangements of the two families we examined in detail, the *SAP *family and the *LIP *family, raise some questions about this model. In only two cases are the most similar family members the closest neighbors (*SAP6 *and *SAP5*; *LIP9 *and *LIP5*). However, the members clustered on one chromosome tend to be most closely related. For the *LIP *gene family, Hube and coworkers [33] showed that *LIP5*, *8*, and *9*, on chromosome 7, form a group and *LIP1*, *2*, *3*, *6*, and *10*, on chromosome 1, are a related but distinct group. *LIP7*, on chromosome R, is an outlier, only distantly related, while *LIP4*, on chromosome 6, fits with the chromosome 7 group. For the *SAP *gene family, *SAP4*, *5*, and *6 *(chromosome 6) form a highly related cluster, while the rest of the group, on chromosomes R, 3, 4, and 6, form a loose association, with the highest similarity being between *SAP2 *on chromosome R and *SAP1*on chromosome 6. These relationships suggest that the families originate on one chromosome and expand there, and when one member is duplicated on another chromosome, the pattern may or may not be repeated. The large number of gene families whose members are dispersed but not randomly would suggest that *C. albicans *is efficient at gene duplication at a distance. However, there are no hints of a specific mechanism in the sequence, such as homology between flanking sequences on different chromosomes or traces of mobile genetic elements. The relatively small number of highly similar ORFs suggests that the gene family members either diverged some time ago or are under strong selection to perform specific functions.

The *TLO *gene family is unique in *C. albicans *because it is found on every chromosome, and there are no closely adjacent members. This suggests that it arose by a mechanism different from, for example, the *LIP *family. One clue is that in all cases it is flanked on its 5' side by the LTR kappa [26]. It seems possible that it has moved via genomic rearrangements caused by the transposon for which kappa is the LTR. An alternative possibility is that this family dispersed by telomere recombination, which is relatively frequent in *S. cerevisiae *[34] and has been shown to occur in *C. albicans *[35]. There are no obvious subtelomeric repeats in *C. albicans*, in contrast to *S. cerevisiae *and *C. glabrata *[36].

The two subgroups of the *TLO *family are differentiated by the presence of an intron. On chromosome 1, there is an interior *TLO *gene, as well as one near each telomere. A plausible explanation for this arrangement is that a chromosome translocation has occurred, with DNA being added to the end of a smaller precursor of chromosome 1, followed by reconstitution of the telomere at the new end generated. There are only three genes in the emerging *C. dubliniensis *sequence with similarity to the *TLO *family, and they are not located at the telomeres. On chromosomes 1 and R in *C. dubliniensis*, the genes adjacent to the *TLO *family member are present and are several kilobases from the end of the assembled sequence, suggesting that the *TLO *gene absence is not due to missing telomere-proximal sequence. Since there are significant differences in virulence between *C. albicans *and *C. dubliniensis*, there may be a role for the *TLO *gene family in some aspect of pathogenesis.

The function of the *TLO *genes is unknown. Although a member of this gene family was isolated as a potential trans-activating protein (and named *CTA2*), based on a one-hybrid screen in *S. cerevisiae*, there is no evidence beyond those experiments as to function [27].

Assembly 21 will be of major importance as studies of the biology and virulence of *C. albicans *continue. It will provide the mapping information that has been lacking due to the absence of a sexual cycle, and it should stimulate experiments in areas as different as evolution and genome dynamics. Among the unsolved questions in the latter area are the detailed structure of the centromere and the function of the MRS. The presence of chromosomal aberrations in clinical isolates was demonstrated early [37,38], and several laboratory strains have recently been shown to be aneuploid [2,3,5]. Genome alterations have recently been shown to play an important role in drug resistance [5], and the complete sequence of each of the chromosomes may lead to the discovery of other changes that affect pathogenesis. Assembly 21 will also be useful for studying aneuploidy in *C. albicans*. Finally, this assembly provides an up-to-date listing of the genes of this important pathogen and will greatly aid its ongoing molecular analysis.

## Materials and methods

### Assembly 21

The first step in Assembly 21 was to assign contigs from Assembly 19 to the appropriate chromosomes. One hundred contigs were anchored by probes on the physical map. To assign the rest, chromosomes were separated by pulse-field gel electrophoresis. Chromosomal bands were eluted from the gel, labeled with either Cy3 of Cy5 dyes, and hybridized to a microarray [10] based on ORFs identified from Assembly 4. As a control, total genomic DNA was also labeled with the reciprocal dye and co-incubated along with the partially purified chromosomes. At least two individual hybridizations (including dye swap controls) were conducted for each of the eight chromosomes. Results and details from these experiments can be seen on our web page [39]. Fluorescence ratios were interpreted visually and it was very easy for us to assign chromosomal localization for 183 Assembly 19 contigs representing 98.4% of DNA sequences and 97.3% of known genes. This analysis also identified nine misassembled contigs by the fact that genes assigned to them hybridized to different chromosomes. The emerging sequence from the closely related species *C. dubliniensis *was then aligned, using megablast, to the Assembly 19 contigs. Phrap was used to connect pairs of contigs that were assigned to the same chromosome and were found to be adjacent based on the *C. dubliniensis *overlap [48]. After the release of the *C. albicans *WO-1 traces, Phrap and these traces were also used to locate misassemblies and to correct them.

The emerging alignments were compared with the physical map and areas of disparity mapped with more probes. Attempts to assemble the contigs into whole chromosomes with Phrap failed because of the large number of repeated elements in the *C. albicans *genome. A custom-made stitcher script (which utilized a 100 nucleotide pure text string starting from the previous alignment) was used to assemble the contig-alignments into chromosomes. The resulting assembly was checked for the presence of all the ORFs from the community annotation [28], and very few ORFs were missing. The penultimate assembly was compared with the optical genome map and the physical map and disparities resolved.

One major discrepancy was the orientation of the sequence between the MRS regions on chromosome 4. The optical map and the physical map showed it in one conformation, while the assembly showed it in the other. The final orientation, consistent with the physical and optical maps, was confirmed by PCR of the border fragments of one MRS and by restriction digestion of genomic DNA and hybridization of a Southern blot with probes from the regions that flank the two MRSs on both sides.

The remaining gaps were filled by PCR followed by sequencing of the products. One gap proved to be the result of a misassembly in a contig. The insertion of a partially overlapping contig closed it. Two gaps were filled by using the draft sequence of Broad Institute's WO-1 assembly [40]. One gap was filled by a sequence from Selmecki *et al*. [5]. The result was called Assembly 20 and it was composed of eight chromosomes with one unresolved (not connected) contig pair. Two gaps were introduced due to the flip of the area between two MRSs on chromosome 4. Two gaps are located around the rDNA region. In addition, most of the MRS regions have not been resolved and can be considered gaps, with the exception of the two MRSs in chromosome 7 and the one MRS in chromosome 6, which were cloned and sequenced at Chiba University.

Following the completion of Assembly 20, we discovered that sequence traces that were used for the assembly and that we had thought were from strain SC5314 were in fact coming from the WO-1 sequencing project. Additional experiments and analysis were thus required to produce Assembly 21 in which the *C. albicans *WO-1 sequences from the contig junctions are replaced with sequences from SC5314. The original traces from SC5314 were obtained from the Stanford Genome Technology Centre and aligned over the junction areas of Assembly 20 using Sequencher 4.7 [41]. Data that were contaminated with WO-1 sequences were tagged as 'Reference sequences', which means that they were not used to construct the consensus sequence for the new alignments. Any missing bases not covered by SC5314 traces were filled with Ns. The resulting alignments can be viewed on our web page [39]. All but 85 junctions could be confidently filled with the SC5314 traces. PCRs were then performed on gaps, overlaps and rearranged regions that had low SC5314 trace coverage. As shown in Additional data file 1, a first round of 85 PCRs was successful in producing 80 amplicons of the expected size and efforts will continue to produce the missing SC5314 sequence data over the next few months. Additional data file 2 includes the sequences of the primers used for these PCR reactions.

### The physical map

A fosmid library was constructed from strain 1161 [42] by M Strathman. Sau3A-digested genomic DNA was inserted into the fosmid vector pFOS1 [43]. The library consists of 3,840 clones with an average insert size of 40 kb (10× genome coverage). For probing, the library was arrayed in 10 384-well plates. Two plates each were printed on a 12 × 16 cm nylon membrane (Hybond-N+, Amersham Pharmacia Biotech Inc Piscataway, NJ, USA), giving a complete library set on five membranes. For printing, freshly thawed fosmid clones were transferred, using a 384-point replicator, to the membrane overlaid on Luria agar containing 20 μg chloramphenicol. The plates were grown overnight at 37°C and the membranes were processed according to the manufacturer's instructions for colony lifts.

Probes were variously clones of genomic DNA, T- and S-end probes from fosmids [44], and PCR products generated from SC5314 DNA using primers based on the public genomic sequences. Probes were randomly labeled with ^32^P. The mapping was carried out as described in Chibana *et al*. [8]. Briefly, membranes containing the library were hybridized with probes and fosmids hybridizing with the same probe were considered to overlap. Probes were also hybridized to Southern blots of pulse-field separations of chromosomes and genomic *Sfi*I digests. The overlapping fosmids were arranged in a linear fashion with chromosome markers like the MRS used to orient the array. The complete set of overlapping fosmids was trimmed to make a minimum tiling set. The physical map is available [45].

### The optical map

The optical map was prepared by OpGen (Madison, WI, USA) using a proprietary technique. The technique involves preparation of large (>500 kb) molecules of genomic DNA, and spreading them on a microfluidic device that causes them to elongate and adhere to the substratum. They are then treated with a restriction enzyme (in this case, *Xho*I). Since the molecules are under slight tension, restriction sites appear as breaks in the molecules. The molecules are then stained with a fluorescent dye, scanned, and the positions of the restriction sites determined, using the amount of fluorescence to calculate the mass of the fragments. The results are averaged over 500 molecules, allowing the preparation of a macro-restriction map. This can be compared with the restriction map derived from the assembly. A more complete description of the technique and more information can be found at [46]. Since the optical map software searches the image database for matches and then constructs the map based on the consensus pattern, the optical map of a chromosome describes only one of the two homologues, and no data on heterozygosity are included.

Although in most cases the three methods were in agreement on the sequence assembly, in cases where disagreements could not be resolved, concurrence of two of the three approaches was deemed sufficient.

The complete Assembly 21 has been submitted to the Candida Genome Database [47], which will be in charge of its maintenance and curation.

## Additional data files

The following additional data are available with the online version of this paper. Additional data file 1 is a figure showing a gel electrophoresis separation of the PCR products produced to close the gaps. Additional data file 2 is a list of the oligonucleotides in the PCR reactions whose products are shown in Additional data file 1.

## Supplementary Material

Additional data file 1 Gel electrophoresis separation of the PCR products produced to close the gaps Click here for file

Additional data file 2 Oligonucleotides in the PCR reactions whose products are shown in Additional data file 1 Click here for file

## Figures and Tables

**Table 1:** Chromosome size and features

	Size (bp)				
				
Chromosome	Assembly 20	Optical map*	Centromere location	MRS	Features
R	2,294,279	2,709,974	1,748,965	1	Ribosomal DNA cluster is ~800 kb on one homologue, ~350 kb on the other. Lacks CARE-2 on the right telomere
1	3,190,598	3,218,448	1,561,879	1	
2	2,233,511	2,228,646	1,924,678	1	The left-hand telomere lacks *TLO *and CARE-2
3	1,798,342	1,794,194	816,770	0	One gap
4	1,622,838	1,619,262	1,000,800	2	
5	1,191,532	1,246,010	465,800	1	
6	1,030,364	1,057,155	975,879	1	
7	943,480	961,199	423,765	2	The right-hand telomere lacks *TLO *and CARE-2

*The MRS is included in all the optical map sizes.

**Table 2:** Chromosome location and similarity of the SAP and LIP gene families

Gene	Chromosome (nearest neighbor)	BLAST score for nearest neighbor	Most closely related member (BLAST)	BLAST score for most closely related member
***SAP *gene family**				
*SAP1*	6 (*SAP4*)	2,453	*SAP2*	3,097
*SAP2*	R (none)	NA	*SAP3*	3,529
*SAP3*	3 (*SAP9*)	326	*SAP2*	3,529
*SAP4*	6 (*SAP1*)	2,453	*SAP6*	5,639
*SAP5*	6 (*SAP6*)	5,100	*SAP6*	5,100
*SAP6*	6 (*SAP5*)	5,121	*SAP4*	5,639
*SAP7*	1 (none)	NA	*SAP6*	637
*SAP8*	3 (*SAP9*)	194	*SAP3*	1,628
*SAP9*	3 (*SAP8*)	194	*SAP10*	296
*SAP10*	4 (none)	NA	*SAP3*	299
***LIP *gene family**				
*LIP1*	1 (*LIP10*)	2,523	*LIP3*	3,976
*LIP2*	1 (*LIP1*)	2,773	*LIP6*	3,320
*LIP3*	1 (LIP6)	2,956	*LIP1*	3,976
*LIP4*	6 (none)	NA	*LIP8*	4,535
*LIP5*	7 (*LIP9*)	4,034	*LIP8*	5,318
*LIP6*	1 (*LIP10*)	2,935	*LIP2*	3,320
*LIP7*	R (none)	NA	*LIP1*	838
*LIP8*	7 (*LIP9*)	2,076	*LIP5*	5,174
*LIP9*	7 (*LIP5*)	4,084	*LIP5*	4,084
*LIP10*	1 (*LIP6*)	2,935	*LIP2*	3,211
