# Supplementary figures and images for: Assembly of the Candida albicans genome into sixteen supercontigs aligned on the eight chromosomes
Source: Genome Biol. 2007 Apr 9;8(4):R52. doi: 10.1186/gb-2007-8-4-r52 (PMC1896002; doi:10.1186/gb-2007-8-4-r52)

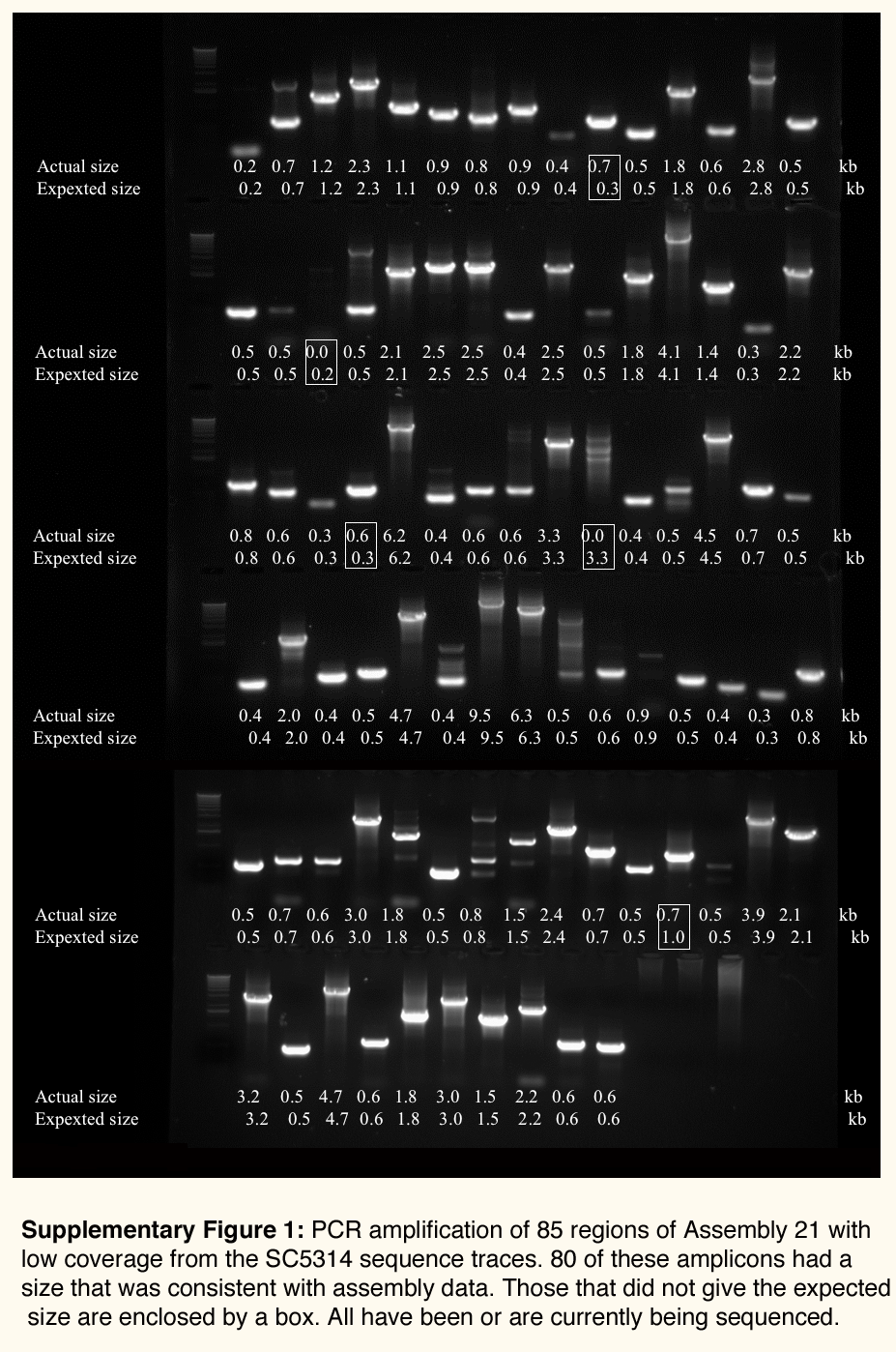

Supplement: Additional data file 1 — Gel electrophoresis separation of the PCR products produced to close the gaps [file gb-2007-8-4-r52-S1.tiff]
